# The Association between *SIRT1* Genetic Variation and Type 2 Diabetes Mellitus Is Influenced by Dietary Intake in Elderly Chinese

**Published:** 2018-09

**Authors:** Zeng ZHUANPING, Liao RIFANG, Chen QING, Chen SIDONG

**Affiliations:** 1. School of Public Health, Guangdong Pharmaceutical University, Guangzhou 510310, China; 2. Dept. of Pharmacy, Sun Yat-Sen Memorial Hospital, Sun Yat-sen University, Guangzhou 510120, China; 3. Dept. of Epidemiology, School of Public Health and Tropical Medicine, Southern Medical University, Guangzhou 510515, China

**Keywords:** Sirtuin 1, Type 2 diabetes mellitus, Dietary, China

## Abstract

**Background:**

To examine whether polymorphisms of *SIRT1* and dietary product intake can be implicated in type 2 diabetes mellitus (T2DM).

**Methods:**

In this community-based, case-control study, 568 subjects (284 patients and 284 controls) were enrolled in a community located in northern Guangzhou, China. The four polymorphisms of *SIRT1* (rs4746720, rs10509291, rs2236319, rs10823116) were examined using TaqMan nuclease technology. The dietary data were collected by an inquiring officer through face-to-face method.

**Results:**

The rs4746720 CC+TT genotype had higher risk compared with CT genotype to develop T2DM [odds ratio (OR) =1.42, 95% confidence interval (CI) = 1.02–1.97]. The subjects with rs4746720 CC+TT genotype and eat sugar food over 30g per day increased the risk of T2DM to 2.22(1.21–4.06) times. The subjects with rs4746720 CC+TT genotype and smoking increased the risk of T2DM to 1.65 (1.10–2.47) times. The unhealthy eating habits such as red meat, salty food, use animal fat yielded higher risks of T2DM, the OR of risk of T2DM was 2.89 (1.38–6.01), 2.73 (1.61–4.64) and 27.91(9.24–84.32) respectively. However, the milk, soy, white meat, vegetables and low-salt diet decreased the risk of T2DM, the OR of risk of T2DM was 0.51 (0.29–0.88), 0.43 (0.26–0.74), 0.51(0.32–0.83), 0.21(0.10–0.44), 0.28(0.12–0.65), 0.35(0.21–0.51) respectively.

**Conclusion:**

Variants in *SIRT1* with rs4746720 CC+TT genotype increased the risk of T2DM, especially with the unhealthy eating habits.

## Introduction

The incidence and prevalence of type 2 diabetes mellitus (T2DM) have highly increased in recent 10 years, combined with its long-term side-effects of cardiovascular disease, visual loss and renal failure, the disease burden of T2DM is serious (1, 2). T2DM was thought to be a disease related to genes and environmental risk factors (3–6), but the pathophysiology of T2DM still not been thoroughly investigated.

The Sirtuin 1 (*SIRT1*) gene belonged to the mammalian Sirtuin family. *SIRT1* was down-regulated in several cells and tissues in insulin-resistant or glucose intolerance states (7). Furthermore, *SIRT1* was regulated by stress and nutritional status (8). In recently, *SIRT1* also regulates adiponectin secretion and glucose production, several *SIRT1* activators have been demonstrated to have beneficial effects on glucose homeostasis and insulin sensitivity in animal models of insulin resistance (9). Decreased *SIRT1* activity may contribute to the development of cat’s T2DM but maybe influenced by nutrient state(10). Therefore, *SIRT1* is an important regulator of energy metabolism. These findings led to a proposed role for *SIRT1* activation in mimicking dietary. Hence, the relation of *SIRT1* variation, dietary intake andT2DM were deserved to take further research. However, we do not found such research on population. Since dietary nutrients were beneficially related to T2DM risk (11–13) and dietary in population was complex, so the aim of our study was to explore the *SIRT1* gene and food intake influenced on T2DM in Chinese senior population.

## Methods

### Study population

Overall, 568 subjects (284 T2DM, 284 controls) were enrolled from community located in northern Guangzhou, China, which have 10 neighborhoods. The subjects who had a fasting serum glucose level ≥7.0 mmol/L were assigned in the case group. Those with a fasting serum glucose level of 3.1–6.0 mmol/L were assigned in the control group. None of the participating subjects were previously diagnosed with T2DM or Impaired Glucose Regulation (IGR). All the subjects came from the same neighborhood, at the same time period. None of the subjects had a history of cancer or digestive diseases. None of the subjects had siblings. The subjects were excluded if they had chronic inflammation, acute disease, other metabolic diseases, or infectious disease. The blood samples and personal information including sex, and year of acquiring T2DM were collected from each participant. The subjects’ body weights and heights were measured and recorded by a nurse.

The study was approved by the Ethics Committee of the first Hospital of Guangdong Pharmaceutical University. Written consent was obtained from all participating subjects.

### Laboratory measurements

The blood samples (2 ml for each) from the vein were immediately placed on ice and separated into plasma and cells within 30 min, then distributed in aliquots and stored at −80 °C until analysis. Genomic DNA was isolated from 0.5 ml blood cells using the approved guideline of the Blood Genomic DNA Purification Kit (Qiagen, Shanghai, China). *SIRT1* gene (rs4746720, rs10509291, rs10823116, and rs2236319) polymorphisms were genotyped using the TaqMan real-time polymerase chain reaction (PCR) assay (Applied Biosystems, CA) without knowledge of the case or control status of the subjects. The TaqMan® Assay primers and FAM/VIC labeled probes by Applied Biosystems (Applied Biosystems, Foster City, CA 4267622). The assay IDs of selected assays were: C-29884088-10 (rs4746720), C-32338526-10 (rs10509291), AH39Y7P (rs10823116), and C-15954063-10 (rs2236319). The ABI Prism 7900HT Sequence Detection System was applied to read the reacted plates and to analyze the endpoint fluorescence. Ten percent of the samples were genotyped in duplicates showing 100% concordance in genotyping results.

Fasting plasma glucose measurements were performed by a specialist using a Beckman Coulter AU680 (Beckman Coulter, Cassina de’ Pecchi, Italy) by glucose oxidase method (14). The lipid and other biochemical parameters were performed by a hospital laboratory (Sun Yat-sen memorial hospital, Sun-Yat-sen University, Guangzhou, China). Plasma cholesterol and TG were quantified by a standardized enzymatic assay (15). We used the standard mercury sphygmomanometer to measure the blood pressure. The staff must participate in the training of standard blood pressure measurement methods prior to the survey. The Body Mass Index (BMI) = Body Weight (kg)÷Height^2 (m).

### Dietary intake data

A questionnaire yielded information on occupation, family history of diabetes, physical examination per year, history of chronic disease, smoking, drinking and dietary data such as consumption of milk, soy, sugary food, vegetables, etc. All the objective data of the study were collected by an inquiring officer through a face-to-face method. We override two questions (drinking milk and eating vegetables) in questionnaire for the reliability test too.

### Statistical Analysis

Cronbach’s Alpha was used to calculate the reliability of the questionnaire. Hardy-Weinberg equilibrium and linkage disequilibrium of the four *SIRT1* gene SNPs were analyzed with the Haploview package. Student’s t-test or χ^2^ test was used to evaluate differences in the distributions of demographic characteristics, selected variables, and genotypes between the cases and control. Fisher exact test was applied when the expected frequency value was less than 5. Body mass index (BMI) was categorized as overweight (BMI≥ 23kg/m^2^) (16) and non-overweight (BMI<23 kg/m^2^) (17).

Further stratified analyses were used to explore the role of the associated polymorphisms in various subgroups. Multiple factor tests were conducted by the use of unconditional Logistical Regression, with an enrollment standardization of 0.05 and discharged by 0.10, backward: conditional. All statistical analyses were performed through the use of SPSS software 12.0 (SPSS, Inc., Chicago, IL).

## Results

### Characteristics of the study populations

There were 568 subjects in our study, 284 were patients and 284 were controls. The average age was 66.23±8.82 (65.64±8.71 in patients, 66.83±8.91 in controls). Overall, 392 subjects enrolled in our study were female (69.0% in patients, 69.0% in controls). 72.01% of subjects had married (70.77% patients, 73.27% controls). 38.03% were educated beyond primary school (35.56% patients, 40.49% controls). We found no significant difference in aspects such as age, sex, marital status, and education between the cases and the controls (*P* values were 0.109, 0.536, 0.513, and 0.226) (Table 1).

**Table 1:** Characteristics of the subjects

***Variables***	***All (n=568)***	***T2DM (n=284)***	***Control (n=284)***	***P***
Age(yr)	66.23±8.82	65.64±8.71	66.83±8.91	0.109
Sex(female)	392(69.0)	196(69.0)	196(69.0)	0.536
Married(N(%))	409(72.01)	201(70.77)	208(73.24)	0.513
Over primary education (N(%))	216(38.03)	101(35.56)	115(40.49)	0.226

### Clinical characteristics of the study population

Compared with control, higher levels of triglyceride, cholesterol, blood pressure, and BMI subjects had higher risk of T2DM, the OR were 3.52(2.48–5.00), 1.95(1.33–2.86), 2.41(1.32–3.38) and 3.78(2.67–5.34) respectively (Table 2).

**Table 2:** Clinical characteristics and type 2 diabetes mellitus

***Variables***	***All N(%)***	***T2DM N(%)***	***Control N(%)***	***OR(95%C.I.)***	***P***
Triglyceride normal	330(58.10)	123(43.31)	207(72.89)	1.00	0
High triglyceride	238(41.90)	161(56.69)	77(27.11)	3.52(2.48–5.00)	
Total cholesterol normal	420(73.94)	192(67.61)	228(80.28)	1.00	0.001
High total cholesterol	148(26.06)	92(32.39)	56(19.72)	1.95(1.33–2.86)	
BMI<23(kg/m^2^)	283(49.82)	96(33.80)	187(65.85)	1.00	0
BMI≥23(kg/m^2^)	285(50.18)	188(66.20)	97(34.15)	3.78(2.67–5.34)	
Blood pressure normal	259(45.60)	99(34.86)	160(56.34)	1.00	0
High blood pressure	309(54.40)	185(65.14)	124(43.66)	2.41(1.32–3.38)	

Triglyceride normal=the level of triglyceride is at 0.56–1.71mmol/L ///High triglyceride=the level of triglyceride is >1.71mmol/L

Total cholesterol normal=the level of total cholesterol is at 3.1–6mmol/L //High total cholesterol=the level of total cholesterol is>6mmol/L)

Blood pressure normal=contractive pressure is <140mmHg and diastolic blood pressure is <90mmHg

High blood pressure=contractive pressure is ≥140mmHg or diastolic blood pressure is ≥90mmHg

### Reliability test of the questionnaire

In the questionnaire, the items were the facts of subjects’ daily lives. We examined two items of the questionnaire (drinking milk and eating vegetables). The Cronbach’s Alpha was 0.745 for drinking milk and 0.617 for eating vegetables. The questionnaire was deemed valid through calculations in the reliability of these two items.

### Behavioral factors of individuals and T2DM

Among the participating seniors, 25.80% of patients and 25.44% of the controls were drinkers; therefore, there was no significant difference in the drinking distribution between the control and the case (*P*=0.923) (Table 3). However, the frequency of physical examination and history of chronic disease was associated with occurrence of T2DM, the *P* values were 0.000 and 0.012.

**Table 3:** The relationship between individual behavior, food factors and type 2 diabetes mellitus

***Variables***		***All (n=568)***	***T2DM (n=284)***	***Control (n=284)***	***P***
Drink	<15ml/d	421(74.38)	210(74.20)	211(74.56)	0.923
	≥15ml/d	145(25.62)	73(25.80)	72(25.44)	
Smoke	no	379(66.84)	189(66.55)	190(67.14)	0.882
	yes	188(33.16)	95(33.45)	93(32.86)	
Physical examination	1times/year	141(24.96)	91(32.04)	50(17.79)	0
	little	138(24.42)	73(25.70)	65(23.13)	
	no	286(50.62)	120(42.25)	166(59.07)	
History of chronic disease	no	368(64.90)	170(59.86)	198(69.96)	0.012
	yes	199(35.10)	114(40.14)	85(30.04)	
Family history of type 2 diabetes mellitus	no	492(86.77)	245(86.27)	247(87.28)	0.722
	yes	75(13.23)	39(13.73)	36(12.72)	
Sugary	≤30g/d	388(68.31)	206(72.54)	182(64.08)	0.03
	>30g/d	180(31.69)	78(27.46)	102(35.92)	
Salty	≤6g/d	204(35.92)	98(34.51)	106(37.32)	0.484
	>6g/d	364(64.08)	186(65.49)	178(62.68)	
Pickled food	≤3 times/w	480(84.51)	252(88.73)	228(80.28)	0.005
	>3 times/w	88(15.49)	32(11.27)	56(19.72)	
Peppery food	≤3 times/w	539(94.89)	275(96.83)	264(92.96)	0.036
	>3 times/w	29(5.11)	9(3.17)	20(7.04)	
Milk	≤200ml/d	397(69.89)	215(75.70)	182(64.08)	0.003
	>200ml/d	171(30.11)	69(24.30)	102(35.91)	
Soy	≤200ml/d	355(62.5)	188(66.20)	167(58.80)	0.069
	200ml/d	213(37.5)	96(33.80)	117(41.20)	
Taste	tasteless	227(39.96)	137(48.24)	90(31.69)	0
	Normal	224(39.44)	77(27.11)	147(51.76)	
	Salt taste	117(20.60)	70(24.65)	47(16.55)	
Vegetables	Little	56(9.86)	33(11.62)	23(8.10)	0
	250g/d	285(50.18)	113(39.79)	172(60.56)	
	251–500g/d	116(20.42)	61(21.48)	55(19.37)	
	Over 500g/d	111(19.54)	77(27.11)	34(11.97)	
Fruit	≤200g/d	147(25.88)	81(28.52)	66(23.24)	0.151
	200ml/d	421(74.12)	203(71.48)	218(76.76)	
Animal fat	Peanut oil	524(92.25)	246(86.62)	278(97.89)	0
	Animal fat	44(7.75)	38(13.38)	6(2.11)	
Red meat	≤40g/d	60(10.58)	24(8.45)	36(12.72)	0.098
	>40g/d	507(89.42)	260(91.55)	247(87.28)	
White meat	≤40g/d	277(48.85)	160(56.34)	117(41.34)	0
	>40g/d	290(51.15)	124(43.66)	166(58.66)	
Viscera	≤20g/d	518(91.36)	265(93.31)	253(89.40)	0.098
	>20g/d	49(8.64)	19(6.69)	30(10.60)	
Fish	≤40g/d	295(52.03)	151(53.17)	144(50.88)	0.586
	>40g/d	272(47.97)	133(46.83)	139(49.12)	
Egg	<1egg/d	309(54.50)	146(51.41)	163(57.60)	0.139
	≥1 egg/d	258(45.50)	138(48.59)	120(42.40)	

Smoke=≥1/day, consecutively 6 months //Little physical examination=have done physical examination, but less than 1 times per year //No physical examination=have not done physical examination.

### Dietary intake factors and T2DM

There were 78 (27.46%) individuals who ate sugary food in the patient group and 102 (35.92%) in the control group (*P*=0.030). Furthermore, the associations between consumption of pickled food, peppery food, or milk to T2DM were tested and the *P* values were 0.005, 0.036 and 0.003, respectively (Table 3).

### Risk association with individual SNP

All SNPs were fitted with the Hardy-Weinberg equilibrium. The allelic distribution of the rs4746720 SNP was significantly different between the case and control (Table 4). As compared to control, the rs4746720 CT genotypes were lower in the case subjects than in the control subjects (46.48% versus 55.28%), *P*=0.036.

**Table 4:** Prevalence of *SIRT1* gene SNPs among the participants

***Models***	***Genotype frequency***	***All (n=568)***	***T2DM (n=284)***	***Control (n=284)***	***P***
	*r*s4746720				
Additive model	TT	177(31.16)	93(32.75)	84(29.58)	0.077
	CT	289(50.88)	132(46.48)	157(55.28)	
	CC	102(17.96)	59(20.77)	43(15.14)	
Recessive model	TT	177(31.16)	93(32.75)	84(29.58)	0.415
	CT+CC	391(68.84)	191(67.25)	200(70.42)	
Dominant model	TT+CT	466(82.04)	225(79.23)	241(84.86)	0.08
	CC	102(17.96)	59(20.77)	43(15.14)	
Co-dominant model	CT	289(50.88)	132(46.48)	157(55.28)	0.036
	TT+CC	279(49.12)	152(53.52)	127(44.72)	
	rs2236319				
Additive model	AA	282(49.65)	144(50.70)	138(48.59)	0.881
	AG	231(40.67)	113(39.79)	118(41.55)	
	GG	55(9.68)	27(9.51)	28(9.86)	
Recessive model	AA	282(49.65)	144(50.70)	138(48.59)	0.615
	AG+GG	286(50.35)	140(49.30)	146(51.41)	
Dominant model	AA+AG	513(90.32)	257(90.49)	256(90.14)	0.887
	GG	55(9.68)	27(9.51)	28(9.86)	
Co-dominant model	AG	231(40.67)	113(39.79)	118(41.55)	0.669
	AA+GG	337(59.33)	171(60.21)	166(58.45)	
	rs10509291				
Additive model	TT	285(50.18)	149(52.46)	136(47.89)	0.426
	AT	232(40.85)	113(39.79)	119(41.90)	
	AA	51(8.98)	22(7.75)	29(10.21)	
Recessive model	TT	285(50.18)	149(52.46)	136(47.89)	0.275
	AT+AA	283(49.82)	135(47.54)	148(52.11)	
Dominant model	TT+AT	517(91.02)	262(92.25)	255(89.79)	0.304
	AA	51(8.98)	22(7.75)	29(10.21)	
Co-dominant model	AT	232(40.84)	113(39.79)	119(41.90)	0.609
	TT+AA	336(59.15)	171(60.21)	165(58.10)	
	rs10823116				
Additive model	AA	223(39.26)	116(40.85)	107(37.68)	0.334
	AG	251(44.19)	117(41.20)	134(47.18)	
	GG	94(16.55)	51(17.96)	43(15.14)	
Recessive model	Aa	223(39.26)	116(40.85)	107(37.68)	0.439
	AG+GG	345(60.74)	168(59.15)	177(62.32)	
Dominant model	AA+AG	474(83.45)	233(82.04)	241(84.86)	0.366
	GG	94(16.55)	51(17.96)	43(15.14)	
Co-dominant model	AG	251(44.19)	117(41.20)	134(47.18)	0.151
	AA+GG	317(55.81)	167(58.80)	150(52.82)	

The rs4746720 CC+TT genotype had higher risk to develop T2DM, compared with CT genotype.

### Subgroup Analyses

Further, we performed stratification analyses for *SIRT1* to explore the role of the polymorphism in the subgroup population (Table 5). For rs4746720, subjects with high triglyceride harboring the CC or TT genotype had a significantly increased risk of T2DM (OR 1.85; 95% CI: 1.06–3.23), compared with subjects of the CT genotype. In red meat more group, the CC or TT genotype was significantly increased T2DM risk (OR 1.43; 95% CI: 1.01–2.02), compared with the subjects of the CT genotype. In sugary food or smoking subjects group, individuals with the CC or TT genotype of rs4746720 had a significantly increased risk of T2DM, compared with individuals carrying the CT genotype (OR 2.22; 95% CI: 1.21–4.06, and OR 1.65; 95%CI: 1.10–2.47).

**Table 5:** Subgroup analysis of rs4746720 and type 2 diabetes mellitus

***Variables***	***OR***	***95.0% C.I.***	***P***
BMI<23(kg/m^2^)	1.61	0.98–2.65	0.059
BMI≥23(kg/m^2^)	1.23	0.75–2.01	0.402
Triglyceride normal	1.33	0.85–2.08	0.211
High triglyceride	1.85	1.06–3.23	0.030
Total cholesterol normal	1.30	0.88–1.91	0.181
High total cholesterol	1.92	0.98–3.78	0.058
Blood pressure normal	1.50	0.91–2.49	0.112
High blood pressure	1.37	0.87–2.16	0.179
Eat red meat little(≤40g/d)	1.12	0.39–3.22	0.830
Red meat more(>40g/d)	1.43	1.01–2.02	0.046
Eat white meat little(≤40g/d)	1.33	0.83–2.15	0.237
White meat more(>40g/d)	1.51	0.95–2.41	0.084
Eat sugar food normal(≤30g/d)	1.19	0.80–1.78	0.389
Sugar food(>30g/d)	2.22	1.21–4.06	0.010
Smoking	1.65	1.10–2.47	0.016
No smoking	1.05	0.59–1.85	0.879

### Multiple Factor Tests

The multiple factor tests (Table 6) showed that milk, soy, white meat, vegetables (0.25 kg to 0.5 kg per day) and low-salt diet decrease the risk of T2DM, with OR of 0.51 (0.29–0.88), 0.43 (0.26–0.74), 0.51 (0.32–0.83), 0.21 (0.10–0.44), 0.28 (0.12–0.65), 0.35 (0.21–0.51) respectively. Red meat, salty food, BMI≥23 kg/m^2^, and the use of animal fat, was associated with a higher risk of T2DM, the OR were 2.89 (1.38–6.01), 2.73 (1.61–4.64), 3.47 (2.28–5.28) and 27.91(9.24–84.32) respectively. The occurrence of rs4746720CC+TT compared with rs4746720CT also had a higher risk of T2DM, with OR 1.61(1.06–2.44). By logistic regression analysis, we did not find two-factor interactions between food consumption, high BMI, and the four SNPs of *SIRT1*.

**Table 6:** Multiple test of *SIRT1* variants, dietary product and type 2 diabetes mellitus

***Variables***	***OR***	***95.0% C.I.***	***P***
Taste(normal vs others)	0.35	0.21–0.57	0.00
Taste(Salt taste vs others)	0.71	0.39–1.31	0.27
Vegetables (250g /d vs others)	0.21	0.10–0.44	0.00
Vegetables (251–500g/d vs others)	0.28	0.12–0.65	0.00
Vegetables (>500g/d vs others)	1.59	0.67–3.74	0.29
Animal fat (animal fat vs peanut oil)	27.91	9.24–84.32	0.00
Red meat (>40g/d vs ≤40g/d)	2.89	1.38–6.07	0.00
White meat (>40g/d vs ≤40g/d)	0.51	0.32–0.83	0.01
Salt food (>30g/d vs ≤30g/d)	2.73	1.61–4.64	0.00
Pickled food (>3 times /w vs ≤3 times/w)	0.16	0.08–0.33	0.00
Peppery food (>3 times /w vs ≤3 times/w)	0.26	0.09–0.75	0.01
Milk(>200ml/d vs ≤200ml/d)	0.51	0.29–0.88	0.02
Soy(>200ml/d vs ≤200ml/d)	0.43	0.26–0.74	0.00
BMI(≥23kg/m^2^ vs <23kg/ m^2^)	3.47	2.28–5.28	0.00
rs4746720(TT+CC vs CT)	1.61	1.06–2.44	0.03

## Discussion

*SIRT1* has been reported to involve of regulating gluconeogenesis and lipogenesis in various tissues (18–20). In the current study, we found *SIRT1* gene rs4746720 plays a dominant role in the pathogenesis of T2DM. Individuals harboring rs4746720CT has a lesser chance of developing the disease than individuals with the rs4746720CC+TT genotype.

The result is biologically plausible because *SIRT1* was considered to be a master regulator involved in several energy homeostasis pathways. The two tag SNPs of *SIRT1* (rs10509291 and rs7896005) were nominally associated with T2DM (21). The SNPs were different between Chinese and Indians because of the difference in race. Furthermore, the *SIRT1* was also studied in vivo.

The role of *SIRT1* in glucose-stimulated insulin secretion may be due to *SIRT1* in pancreatic beta cells which significantly improved glucose tolerance, and enhanced insulin response to glucose. In our research, we performed stratification analyses for *SIRT1* to explore the role of the polymorphism in the subgroup population (Table 5).For rs4746720, subjects harboring the CC or TT genotype had a significantly increased risk of T2DM compared with the CT genotype, especially in which have high triglyceride, eat more red meat, with sugar food and smoking. This result indicated the interaction of red meat and the rs4746720 CC+TT genotype and both factors had additive effect on the risk of diabetes development. However, by logistic regression analysis, we did not found two-factor interactions between food consumption and the four SNPs of *SIRT1*. Further large sample research is needed to determine the effect of these factors.

The milk and some dietary products were associated with a lower risk of T2DM. Some components in dietary products, such as lactose and dairy protein, may enhance satiety and reduce the risk of obesity (risk factors for T2DM) relative to other high-carbohydrate foods and beverages (22). The soy and white meat intake reduce the risk of T2DM was also revealed in this study. However, a recent evaluation of clinical trials that assessed the effect of dairy products or calcium intake on adiposity, with or without concomitant energy restriction, did not support this hypothesis (22, 23). We identified in Chinese old population that vegetable consumption was associated with lower risk of diabetes. This association was independent of age, current smoking status, alcohol intake, and family history of diabetes. However, no significant associations were observed between intake of fruits and the development of diabetes. The protective effects of vegetables on the development of diabetes could be attributed to their antioxidant properties, as well as to their dietary fiber and Mg content (24, 25). Our results are in agreement with these studies reporting that the more vegetable lifestyle was associated with reduced risk of diabetes. However, the evidence regarding the role of vegetable intake in relation to diabetes risk has remained inconclusive. Eating vegetables may generally be an indicator of a more health-conscious attitude; therefore, a diet rich in vegetables may correspond to reduced risk of diabetes. However, T2DM is a polygenic disease and fruits may still play some roles in the disease, although we could not distinguish any effect.

In a Canadian cross-sectional study, researchers found that a pattern characterized by heavy consumption of French fries, chocolate, cake, canned meat, and canned fruit was associated with a higher prevalence of type 2 diabetes (26). We also found that red meat, salty food, BMI≥23, and use of animal fat will increase the risk of T2DM. These results demonstrated a considerably lower risk of diabetes among the intervention group prescribed healthy diet and exercise. It is the first time we found that pickled and pungent foods decreased the risk of T2DM. The pickled and pungent foods are popular food in southern China. These foods contain abundant vitamins and minerals, which would serve to decrease the risk of T2DM.

The population in this study was limited in diversity, thus the results probably have little genetic drift. Our study population consisted only of elderly Han people, so the results may not apply to other populations. At same time, the questionnaire asked subjects to recall food intake, the recall bias cannot be fully excluded.

## Conclusion

Our study highlights the contribution of *SIRT1*, the rs4746720 CC+TT genotype and unhealthy dietary intake to the development of T2DM. Further population-based or cohort studies are needed to confirm these results.

## Ethical considerations

Ethical issues (Including plagiarism, informed consent, misconduct, data fabrication and/or falsification, double publication and/or submission, redundancy, etc.) have been completely observed by the authors.
